# Identifying the know-do gap in evidence-based neonatal care practices among informal health care providers—a cross-sectional study from Ujjain, India

**DOI:** 10.1186/s12913-020-05805-2

**Published:** 2020-10-21

**Authors:** Isaac Gikandi Mungai, Sumit Singh Baghel, Shuchi Soni, Shailja Vagela, Megha Sharma, Vishal Diwan, Ashok J. Tamhankar, Cecilia Stålsby Lundborg, Ashish Pathak

**Affiliations:** 1 Department of Global Public Health—Health Systems and Policy (HSP): Medicines Focusing Antibiotics, Karolinska Institutet, 17177 Stockholm, Sweden; 2 Department of Pediatrics, RD Gardi Medical College, Ujjain, MP 456010 India; 3 Department of Pharmacology, RD Gardi Medical College, Ujjain, MP 4560101 India; 4 National Institute for Research in Environmental Health (NIREH), Bhopal, MP India; 5 Indian Initiative for Management of Antibiotic Resistance, Department of Environmental Medicine, R.D. Gardi Medical College, Ujjain, 456006 India; 6 Department of Women and Children’s Health, International Maternal and Child Health Unit, Uppsala University, SE-751 85 Uppsala, Sweden

**Keywords:** Informal healthcare providers, Knowledge, Neonatal care, Evidence-based practice, India

## Abstract

**Background:**

More than a quarter of global neonatal deaths are reported from India, and a large proportion of these deaths are preventable. However, in the absence of robust public health care systems in several states in India, informal health care providers (IHCPs) with no formal medical education are the first contact service providers. The aim of this study was to assess the knowledge of IHCPs in basic evidence-based practices in neonatal care in Ujjain district and investigated factors associated with differences in levels of knowledge.

**Methods:**

A cross-sectional survey was conducted using a questionnaire with multiple-choice questions covering the basic elements of neonatal care. The total score of the IHCPs was calculated. Multivariate quantile regression model was used to look for association of IHCPs knowledge score with: the practitioners’ age, years of experience, number of patients treated per day, and whether they attended children in their practice.

**Results:**

Of the 945 IHCPs approached, 830 (88%) participated in the study. The mean ± SD score achieved was 22.3 ± 7.7, with a median score of 21 out of maximum score of 48. Although IHCPs could identify key tenets of enhancing survival chances of neonates, they scored low on the specifics of cord care, breastfeeding, vitamin K use to prevent neonatal hemorrhage, and identification and care of low-birth-weight babies. The practitioners particularly lacked knowledge about neonatal resuscitation, and only a small proportion reported following up on immunizations. Results of quantile regression analysis showed that more than 5 years of practice experience and treating more than 20 patients per day had a statistically significant positive association with the knowledge score at higher quantiles (q75^th^ and q90th) only. IHCPs treating children had significantly better scores across quantiles accept at the highest quantile (90^th^).

**Conclusions:**

The present study highlighted that know-do gap exists in evidence-based practices for all key areas of neonatal care tested among the IHCPs. The study provides the evidence that some IHCPs do possess knowledge in basic evidence-based practices in neonatal care, which could be built upon by future educational interventions. Targeting IHCPs can be an innovative way to reach a large rural population in the study setting and to improve neonatal care services.

## Background

Globally, under-5 mortality (U5M) decreased by 53% during the Millennium Development Goals era between 1990 and 2015, from 91 to 43 per 1000 live births, mainly due to reduction in post neonatal deaths [1]. During the same period, global neonatal mortality declined by 47%, from 36 to 19 per 1000 live births, but still accounted for 45% of the total U5M [1]. New targeted strategies are required to further reduce the neonatal mortality [2]. The South Asian region contributed to approximately 1 million deaths in 2015 [3], with approximately 3000 deaths per day in the region, despite a 51% decrease in neonatal mortality from 1990 to 2015 [3]. India, Pakistan, and Afghanistan have made the slowest progress in reducing neonatal mortality rates (NMRs) [4]. India contributes to one-fifth of global live births and more than a quarter of neonatal deaths [3, 5]. The current NMR in India is 24 per 1000 live births and U5M rate is 39 per 1000 live births; thus, more than 60% of U5M is observed in the neonatal period [6]. Uttar Pradesh, Madhya Pradesh (MP), Bihar, and Rajasthan contribute to approximately 55% of the total neonatal deaths in India [4, 6]. Moreover, NMR in rural areas is twice as that in urban areas (27 versus 14 per 1000 live births) [6].

A bottleneck analysis revealed that the absence of skilled human resource, service delivery issues, insufficient financial resources, and lack of community ownership are the key barriers in effective scale-up of neonatal interventions to reduce NMRs [7]. The aforementioned factors coupled with the fact that approximately 71% of health care in India was financed out of pocket, leading to destitute and impoverishment in 18% Indian households [8]. Gaps in the coverage and quality of services in the public health sector result in the rural population seeking health care from informal health care providers (IHCPs). IHCPs are a large group of health care providers who have not received any formally recognized training with a defined curriculum, operate outside of institutional regulations, and receive payments directly from their clients [9]. They are the main health care providers for the poor in rural MP and Ujjain district, where the present study was conducted. Because IHCPs are an existing part of the health care system, using them to tackle NM and to attain desirable outcomes in neonatal care could be beneficial. Educational interventions, such as capacity-building exercises and training programs, have been recommended for IHCPs to improve the health care outcome [9]. The aim of this study was to assess the knowledge of IHCPs in basic evidence-based procedures in neonatal care in Ujjain district and an also to investigate factors associated with differences in levels of knowledge. The findings of the study will act as a reference point to future targeted education intervention.

## Methods

### Study design and study setting

This cross-sectional survey was conducted in Ujjain district (6091 km^2^), located in the western part of the state of MP, that has a population of 1.9 million [10]. It is administratively subdivided into seven tehsils or subdistricts [11]. The MP state ranked last among states in the Inequality-adjusted Human Development Index with an index of 0.29 in 2011 [12]. Ujjain district has a large rural population (73%) and high poverty level with 37% of population living below the poverty line [12]. According to the Census of India, Annual Health Survey Report, the NMRs of MP, and Ujjain district were 41 per 1000, and 33 per1000 live births, respectively, which is higher than the national average of 24 per1000 live births [11].

In the state of MP the unqualified health care providers-IHCPs are more likely to be working in rural and less economically endowed regions [13]. A low-qualified provider density (< 2.5/1000) was observed for the 41 districts of MP [13]. A study that mapped private providers in Ujjain district also observed that the density of unqualified private providers was higher in rural areas (1:968) than in urban areas (1:8279) [14].

### Questionnaire for data collection

Data were collected using a questionnaire which consisted of 16 multiple-choice questions covering the following essential newborn care practices: breastfeeding practices; care of low-birth-weight neonates, including neonatal hypothermia; infection prevention by correct cord care (allowing it to dry) and following up on immunizations; prophylactic administration of vitamin K in right doses (1 mg) and calculating doses of medicines according to the weight of the child; and using bag and mask ventilation in neonates who do not start breathing spontaneously after birth. The questionnaire was adopted from a similar survey, but questions related to home visit of health care workers in the neonatal period were not included to adopt for local context [15]. The questionnaire was translated to Hindi, the local language, by two independent translators: one a subject expert and the other an expert in Hindi language, according to the World Health Organization (WHO) methodology [16] (Questionnaire in Hindi and English-Additional file 1).

### Pilot testing of questionnaire

A pilot study was conducted on 56 IHCPs for testing the questionnaire and calculating the sample size. Based on the feedback from the pilot study, one question regarding the IHCP qualification was omitted because it was perceived as a potential evidence for “illegality” of clinical practice by participating IHCPs. The participants of the pilot study correctly answered 45% of the questions. Participants of the pilot study were not included in the main study.

### Sample size calculation

Based on pilot study the sample size calculation was done assuming that IHCPs can correctly answer 45% of questions included in the questionnaire. Thus, to detect at least a 5% difference around the proportion of 0.45 with power of 80%, two-sided alpha of 0.05, the minimum sample size required was 780. Assuming a non-response rate of 20%, we decided to approach 945 IHCP’s to achieve the desired sample size.

### Sampling frame and data collection

**Fig. 1:**
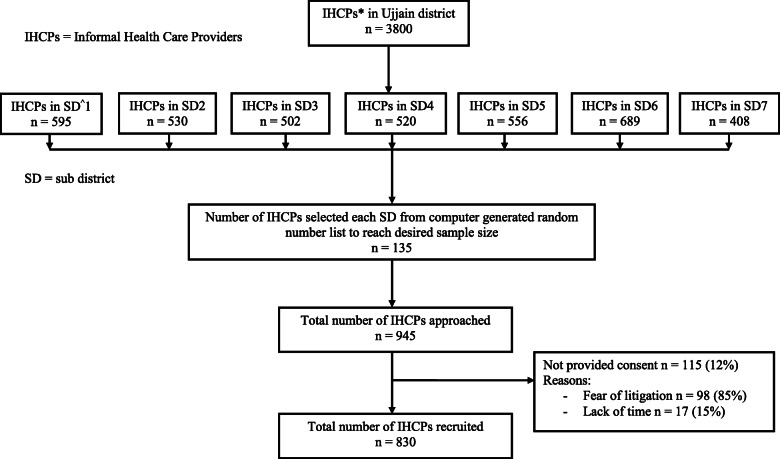
Flow chart explaining participant recruitment procedure

A previous study, done in 2004 identified and mapped using geographical information systems approximately 1162 IHCPs in seven sub districts or tehsils of Ujjain district [14]. In 2013 an association of unqualified/unregistered doctors was formed, which stated the number of IHCPs in Ujjain district as 3800 and provided a sub district wise list, which was used for selecting 135 IHCPs per subdistrict by simple random sampling by computer generated numbers. The number of IHCPs were not verified before sampling. Thus, 945 IHCPs were approached, and 830 provided consent to participate in the study (response rate = 88%). Those who refused to participate cited fear of litigation and lack of time. Figure 1 provides flow chart of participant recruitment procedure. Six volunteers trained for data collection and with a significant understanding of the local area and language visited 945 IHCPs for 2 months in 2014 to complete the questionnaires. The visits were scheduled as per the convenience of IHCPs to avoid rush hours of patient consultation. The entire questionnaire had to be completed by IHCPs in the presence of the data collector. This was done to explain the correct meaning of the question if the participant had any difficulty in understanding. No inducements were given to the IHCPs for participation in the study.

### Outcome measure

The main outcome measure (dependent variable) was a knowledge score of various aspects of neonatal care. Independent variables described the IHCPs in terms of: a) age of the respondents b) duration of practice in years c) approximate number of patient seen per day- The choices included 5–10, 10–20, 20–40 and more than 50. d) whether they treated children or not at the time of study. All the IHCPs that were not treating children at the time of the study indicated they would do so in near future. Thus, they were included in the study.

### Statistical analysis

A knowledge score was calculated for each respondent. Each respondent had to select 16 correct choices to achieve the maximum score. One correct choice generated a maximum of 3 points, whereas 1 point was reduced for incorrect alternatives. A participant could obtain maximum 48 points. Multivariate quantile regression models were used to test the association between the knowledge scores of IHCPs and independent variables: the characteristics of the practitioners- age (continuous variable), years of practice (more than and equal to 5 versus less than 5) and number of patients seen per day (more than and equal to 20 versus less than 20) and if the practitioner treats children or not currently (no versus yes). Quantile regression model was chosen to capture the full distribution of the outcome- the knowledge scores. The coefficient (b), standard error, and 95% confidence interval (CI) were estimated for 10th, 25th, 50th (median), 75th, and 90th quantiles of the knowledge scores based on 500 bootstrap samples. Analysis was performed using Stata (Version 13.0, StataCorp, College Station, TX, USA).

## Results

### Characteristics of IHCPs

Of 945 IHCPs, 830 (88%) responded to the questionnaires. The mean age of the respondents was 37.5 years (standard deviation (SD) 10.7) with a median age of 36 years. The practitioners had a working experience of 1–52 years, with the mean (±SD) years of practice being 11.5 (±9.3) years and median of 10 years. Majority (77%, *n* = 636) of the practitioners reported consulting 5–20 patients every day. A total of 81% (*n* = 671) practitioners reported offering pediatric consultations. Table 1 presents the characteristics of IHCPs included in the study.

**Table 1:** Characteristics of informal health care providers (*n* = 830) of Ujjain district included in the study

	Frequency (***n*** = 830)	Percentage
**Age of Practitioners (Years)**		
18–30	184	22
> 30–45	494	60
> 45–80	152	18
**Years of practice**		
< 5	187	23
5–10	323	39
> 10	320	39
**Number of patients seen per day**		
5–10	440	53
10–20	236	28
20–40	116	14
> 50	38	5
**Practitioners see children in practice**		
Yes	671	81
No	159	19

### Knowledge survey responses

The mean ± SD score achieved was 22.3 ± 7.7, with a range between 0 and 48 and a median score of 21. Tables 2 and 3 provide the details of IHCP responses to the knowledge questionnaire. Significant results are presented below.

**Table 2:** Results of the evidence-based neonatal care practices knowledge survey among informal health care providers (*n* = 830) of Ujjain district for single answer questions

Statement with correct answer in the bracket	Frequency (***n*** = 830)	Percentage
**Breastfeeding initiation within? (half hour after birth)**		
Correct	572	69
**Exclusive breastfeeding up to? (the age of 6 months)**		
Correct	627	76
**Age of cessation of breastfeeding? (Beyond 2 years of age)**		
Correct	111	13
**Dosage of vitamin k according to national guidelines? (1 mg)**		
Correct	385	46
**Best method of cord care? (Dry cord care)**		
Correct	228	27
**Best practice to control hypothermia? (Kangaroo Mother Care)**		
Correct	529	64
**Definition of a term low birth weight baby. (Birth weight of 2.5 Kg or below)**		
Correct	297	36
**Whether practitioner takes weight of babies? (Yes)**		
Yes	74	9
**Should children be given medicine according to their weight? (yes)**		
Yes	765	92
**Whether practitioner asks about immunization to children visiting them? (yes)**		
Yes	184	22

**Table 3:** Results of the evidence-based neonatal care practices knowledge survey among informal health care providers (*n* = 830) of Ujjain district where multiple responses could be given

Statement	Frequency (***n*** = 830)	Percentage
**Advice when mother is not having enough milk**		
Give top-up milk (bottle-feeding)	231	26
Increase frequency of breastfeeding	436	50
Give jiggery water, natural herbs, honey etc. Until the mother starts getting milk	100	11
Advise mother to breastfeed the baby by other mothers having enough milk	71	8
Not known	33	4
Others	8	1
**Neonatal resuscitation (best practice if baby has poor respiration)**		
Wipe with clean clothes	120	13
Give ventilation with bag and mask	156	17
Clean mouth and nose if required	126	14
Hold the child by the feet and pat the back	369	41
Pouring cold water on the child	28	3
Not known	77	9
Others	24	3
**To prevent neonatal bleeding**		
Breastfeed child	180	22
No need for any medicine	33	4
Give vitamin k	493	59
Do not know	116	14
Others	14	2
**Important in care of a low birth weight baby**		
Frequent washing of the baby	46	6
Early initiation and frequent breastfeeding	554	67
To keep the baby warm	433	52
Protecting the baby from infection	614	74
Not known	40	5
Others	6	1

Initiating breastfeeding within half an hour of birth and the duration of exclusive breastfeeding for up to 6 months were correctly indicated by 69% (*n* = 572) and 76% (*n* = 627) of IHCPs, respectively. In cases where the mother reported not having enough milk, only 50% (*n* = 436) of the practitioners provided the correct advice to increase the frequency of breastfeeding, and 26% of them advised mothers to proceed with bottle-feeding. Only 13% (*n* = 111) of the practitioners correctly identified that breastfeeding should be ceased only after more than 2 years.

Only 9% (*n* = 70) of the respondents measured weights of the babies. Additionally, only 36% (*n* = 302) of the practitioners correctly identified the cutoff for a low-birth-weight neonate as less than 2500 g. A low-birth-weight baby placed in skin-to-skin contact with the mother (kangaroo care) was considered the best practice to prevent neonatal hypothermia by 65% (*n* = 549) respondents.

A total of 28% (*n* = 235) IHCPs responded correctly that the umbilical cord should be allowed to dry to ensure its best care. Slightly more than half of the respondents (51%) answered that they would apply antibiotic powder. Only 22% (*n* = 184) IHCPs responded that they generally inquired about the immunization status of children they attended. While 59% (*n* = 493) of the respondents recommended prophylactic vitamin K as the best way to prevent neonatal bleeding, only 20% (*n* = 162) correctly identified the appropriate dosage.

Given a vignette in which the respondents were to deduce that the baby had poor respiration after birth, 17% (*n* = 156) correctly identified that they would perform resuscitation using a bag and mask. However, 41% (*n* = 369) IHCPs incorrectly opted to hold the baby by the legs and pat the back. Very few IHCPs correctly identified the initial steps of resuscitation. Only 14% (*n* = 126) knew to clean the mouth and nose, and only 13% (*n* = 120) responded that they would wipe the baby with dry pre-warmed clothes.

### Relationship between the scores and practitioner characteristics

Table 4 shows the results of quantile regression analysis of the participant characteristics with knowledge score. Age of the participants had no significant association with the knowledge score across all percentiles. More than 5 years of practice experience and treating more than 20 patients per day had a statistically significant positive association with the knowledge score only at higher quantiles (q75^th^ and q90^th^) and had no statistically significant association at lower quantiles (q10^th^ and q25^th^) and at median quantile (50th). IHCPs treating children had significantly better scores across quantiles accept at the highest quantile (90th).

**Table 4:** Quantile regression analysis of the participant characteristics with knowledge score of 830 IHCPs in Ujjain, India

	Q10^th^		q25^th^		q50^th^ (median)		q75^th^		q90^th^	
	b (95% CI)	*p* value	b (95% CI)	*p* value	b (95% CI)	*p* value	b (95% CI)	*p* value	b (95% CI)	*p* value
Age (years)*										
	−1.05 (−0.13 to 0.13)	1.000	4.85 (−0.11 to 0.11)	1.000	1.02 (−0.07 to 0.07)	1.000	2.16 (−0.07 to 0.07)	1.000	7.63 (−0.13 to − 0.13)	1.000
Year of experience										
More than 5 (ref) Less than and equal to 5	4 (−0.63 to 8.63)	0.090	4 (1.37 to 6.62)	1.000	−5.55 (−3.96 to 3.96)	1.000	4 (0.55 to 7.44)	0.023	4 (−0.30 to 7.69)	0.034
Number of patients seen per day										
More than 20 (ref) Less than and equal to 20	−1.41 (− 3.49 to 3.49)	1.000	−4 (−7.12 to −0.87)	0.062	0 (− 3.11 to 3.11)	1.000	4 (0.59 to 7.40)	0.021	4 (0.83 to 7.16)	0.013
Practitioner treats children										
No (ref) Yes	8 (3.55 to 12.44)	< 0.001	8 (3.49 to 12.05)	0.001	4 (1.16 to 6.83)	0.006	4 (0.58 to 7.41)	0.022	−4.01 (−2.90 to 2.90)	1.000

*b* Beta coefficient, *CI* Confidence interval, *q* Quantile, *ref*. Reference, % = percentage, * = continuous variable

## Discussion

This study assessed the knowledge gaps of IHCPs in Ujjain district, MP, India, on evidence-based practices of neonatal care. IHCPs presented good knowledge of the initiation and frequency of breastfeeding and identified the best practices in protecting low-birth-weight babies from hypothermia. However, the practitioners lacked knowledge about the duration of exclusive breastfeeding, neonatal resuscitation, the correct dosage for vitamin K in neonatal hemorrhage prophylaxis, umbilical cord care, and follow-up on the immunization status of the children. A know-do gap existed in understanding the importance of measuring weights of the children and practically measuring weights.

Most of the published studies on IHCPs have focused on characterizing the scope of their work, utilization patterns, and reason for their popularity, especially in low-income countries [9]. It is perceived that IHCPs provide substandard care compared with formally trained health care providers. However, not many studies have characterized the knowledge gap among IHCPs. A study conducted in Bangladesh revealed that IHCPs scored lower than allopathic (trained) paramedical professionals on the knowledge of adequate drug provisions [17]. A study conducted in Vietnam revealed irrational dispensing of antibiotics, noncompliance to national guidelines, and increased use of traditional medicines [17]. However, extremely few studies have been conducted on the knowledge gap of IHCPs on evidence-based practice in neonatal care [9]. In a field trial conducted in rural Gadchiroli, India [18], home-based interventions for birth-asphyxia delivered by a team of traditional birth attendants and semiskilled village health workers reduced the asphyxia-related NM by 65% compared with that by only the traditional birth attendants [18]. Since, birth-asphyxia is one of the major cause of neonatal mortality and morbidity and since most of the IHCPs in our study did not do well in identifying the initial steps of resuscitation an intervention similar to the Gadchiroli home-based intervention [18] could yield high dividends in reducing NM.

The duration of breastfeeding was unknown to most IHCPs in the present study; however, it is not well-known even to qualified health care workers [15]. Inadequate knowledge on the use of vitamin K for prophylaxis against neonatal bleeding and the use of bag and mask for resuscitation in the present study was similar to that reported in a study from Vietnam [15]. This could be due to lack of knowledge and skills among IHCPs of basic resuscitation equipment and drugs [15]. However, an obvious know-do gap existed among IHCPs who responded that they do not weigh the neonates despite being aware that dosages of drugs should be based on the weights of the children.

The absence of knowledge of safe umbilical cord care is not unique to our setting. A firm tradition of umbilical cord care has been established in every culture [19]. Cord care practices vary across countries or cultural groups within a country and include a wide range of substances [19]. Since 1998, the WHO advocates using dry umbilical cord care; however, in situations where hygienic conditions are poor and/or infection rates are high, chlorhexidine application is recommended [20]. A total of 51% respondents in our study assumed that antibiotics should be applied to the umbilical cord. Promoting healing and hastening cord separation are the underlying beliefs related to the application of substances to the umbilical cord [19]. Among the IHCPs in our study, antibiotic application could be due to the fear of infection; it also reflects a behavior to use antibiotics irrationally. IHCP’s prescribing behavior, with high rates of antibiotic prescribing has been documented in a recent study in the same area [21].

The response on follow-up on immunization history raises issues regarding provider awareness of the importance of this public health intervention. In 2017, an estimated 19.9 million infants worldwide were deprived of routine immunization services, such as three doses of the diphtheria, tetanus, and pertussis vaccine [22]. India accounts for one-fourth of these under-immunized children [23]. Because IHCPs become the main providers of care where the formal system has failed, the risk factors for incomplete immunization, such as illiteracy among mothers, living in rural areas, belonging to scheduled tribes/castes, and high birth order, all reflecting inequity in immunization coverage [24], are also prevalent among their clients. Because the Government of India has introduced new vaccines in the recent times, IHCPs should be provided with an opportunity to become aware of the immunization services offered by the government, so that they can offer referrals to clients who need an immunization service. Most IHCPs learn prescribing informally from working as assistants to formal healthcare providers and in pharmacies. This can explain the results of the quantile regression analysis which showed that IHCPs with more years of experience, those seeing more patients and those treating children in their practice performed better especially in higher quantiles (Table 4). If education opportunities are provided to them IHCPs could become part of formal healthcare system.

The Helping Babies Survive program, which was implemented in resource constrained settings is an example of skill upgradation of health care workers [25]. It was developed to reduce preventable new born deaths through skill-based learning using simulation, learning exercises, and peer-to-peer training of healthcare providers [25]. A systematic review suggested that there were significant short-term improvements in knowledge and skills scores but results were not sustained over time. However, despite this lack of sustainability of skills there was a significant drop in number of fresh stillbirth and first-day mortality rate decreased [25]. This was primarily attributed to increase use of bag-and-mask ventilation within the golden minute [26].

The Government of India has recently launched its flagship program ‘skill development’ and has also decided to upgrade the skills of IHCPs by providing short-term training or courses [27]. We are hopeful that if appropriate skills are imparted this may help reduce the neonatal mortality in India. This is important in the light of the fact that provision of formally trained health care providers in the rural health care system in India will continue to be poor in the next few years [28], the skill upgradation of IHCPs can prove to be a low hanging fruit to reduce neonatal mortality in India.

### Methodological considerations

The main strength of the study is that the questionnaire used has been used before to assess the level of knowledge on evidence-based neonatal care in Vietnam among qualified primary health care workers the questionnaire used in the present study was pilot tested which strengthens internal validity. Because it was administered in Hindi, the preferred language of the respondents, forward and backward translations were performed with the help of Hindi and English language experts to ensure retained meaning of the questions post translation. A combination of single-answer and multiple-correct-answer questions has been proposed for reducing guesswork [29]; this combination was used this questionnaire.

The study did not record the number of neonates seen by the IHCPs but the total patients. This information could have helped us in identifying targets for future intervention. IHCPs by definition do not possess a regulatory body. The nature of practice is also such that they might be mobile, conducting home visits but also having more than one clinic., The difficulty in developing a sampling frame was compounded by the fact that the IHCPs work in remote areas. A complex relationship of mistrust between private and public providers has been described previously in the state of MP [30], which could have hampered the ability of IHCPs to participate in the study without the fear of reprisal.

## Conclusions

The present study characterized the gap in knowledge of evidence-based practice in neonatal care among IHCPs in Ujjain, MP, India. Less use of evidence-based practice is a cause of high neonatal mortality globally. The study provides the evidence that some IHCPs do possess knowledge in basic evidence-based practices in neonatal care, which could be built upon by future educational interventions. Targeting IHCPs can be an innovative way to reach a large rural population in the study setting and to improve neonatal care services.

## Supplementary information

**Additional file 1.**

## Data Availability

The dataset used and/or analysed during the current study is available from the corresponding author on reasonable request. Individual data can due to confidentiality reasons not be made public. All enquiries regarding data sharing should be made to The Chairman, Institutional Ethics Committee, R D Gardi Medical College, Agar Road, Ujjain, India 456006 (E-mail uctharc@sancharnet.in). The name of data set corresponding to the study is IHCP_Neo_know_study data (IEC approval number 300/2013).

## Abbreviations

IHCPs: Informal health care providers
U-5: Under 5 years
NMR: Neonatal mortality rate
MP: Madhya Pradesh
WHO: World health organization
SD: Standard deviation
OR: Odds ratio
CI: Confidence intervals
