# Supplementary material for: Identifying the know-do gap in evidence-based neonatal care practices among informal health care providers—a cross-sectional study from Ujjain, India
Source: BMC Health Serv Res. 2020 Oct 21;20:966. doi: 10.1186/s12913-020-05805-2 (PMC7576775; doi:10.1186/s12913-020-05805-2)
Supplement: Supplementary file 1 — Additional file 1. [file 12913_2020_5805_MOESM1_ESM.pdf]

R D Gardi Medical College, Ujjain, Madhya Pradesh

Questionnaire for health care workers regarding neonatal care

Section A: General information

**1. What is your age?**	**_______(Years)**
**2. How many years of practice do you have ?**	**_______(Years)**
**3. How many patients do you seen per day?**	
1) 5-10	
2) 10-20	☐
3) 20-40	☐
4) >50	☐
**4. Do you see children in practice?**	
Yes	☐
No	☐

Section B: Neonatal care practices (only single response per question can be given)

**5. At what time breastfeeding should be initiated in neonates?**	
1) Within half hour after birth	☐
2) After 1 to 6 hours of birth	☐
3) After 6 to 12 hours of birth	☐
4) After 12 hours of birth	☐
5) Don’t know	☐
6) Others	☐
**6. For how much exclusive breastfeeding should be given in neonates?**	
1) Till 1 month	☐
2) Till 2 month	☐
3) Till 4 month	☐
4) Till 6 month	☐
5) Don’t know	☐
6) Others	☐
**7. At what age exclusive breastfeeding can be stopped in neonates?**	
1) 6 months	☐
2) 12 months	☐
3) 18 months	☐
4) 24 months	☐
5) After 2 years	☐
6) Don’t know	☐
7) Others	☐
**8. What is the dosage of vitamin k according to national guidelines?**	
1) 0.5 mg	☐
2) 1 mg	☐
3) 5 mg	☐
4) 10 mg	☐

5) Don't know	☐
6) Others	☐
**9. What is the best method of cord care**	☐
1) Allow to dry the cord by its own	☐
2) Regularly clean with soap and water	☐
3) Use antibiotic powder	☐
4) Consult hospital	☐
	☐
5) Don't know	☐
6) Others	☐
**10. What is the best practice to control hypothermia?**	
1) Giving bath to neonate with slightly hot water	☐
2) Keep child in cloths with head covered	☐
3) Keep child attached to mother's skin	☐
4) Keep child in a room with 28-30c temperature	☐
5) Keep child near to a source of heat like heater, fire etc	☐
6) Don't know	☐
7) Others	☐
**11. What is the definition of a term low birth weight baby**	
1) Less than 3000 grams	☐
2) Less than 2500 grams	☐
3) Less than 1500 grams	☐
4) Less than 1000 grams	☐
5) Don't know	☐
6) Others	☐
**12. Do you weigh the child coming to you clinic ?**	
1) Yes	☐
2) No	☐
**13. Do you think children be given medicine according to their weight?**	
1) Yes	☐
2) No	☐
**14. Do you ask about immunization to children**	
1) Yes	☐
2) No	☐

Section C: Neonatal care practices (multiple response per question can be given)

**15. After delivery if mother is complaining that she is not having enough milk then what advice will you give?**	
1) Give top-up milk (bottle-feeding)	☐
2) Increase frequency of breastfeeding	☐
3) Give jiggery water, natural herbs, honey etc. Until the mother starts getting milk	☐
4) Advise mother to breastfeed the baby by other mothers having enough milk	☐
5) Not known	☐
6) Others	☐
**16. What are the best practice for neonatal resuscitation if baby has poor respiration?**	
1) Wipe with clean clothes	☐
2) Give ventilation with bag and mask	☐
	☐
3) Clean mouth and nose if required	☐
4) Hold the child by the feet and pat the back	☐
	☐
5) Pouring cold water on the child	☐
6) Not known	☐
7) Others	☐
**17. What should be done to prevent neonatal bleeding?**	
1) Breastfeed child	☐
2) No need for any medicine	☐
3) Give vitamin k	☐
4) Do not know	☐
5) Others	☐
**18. What is important when providing care to a low birth weight baby?**	
1) Frequent washing of the baby	☐
2) Early initiation and frequent breastfeeding	☐
	☐
3) To keep the baby warm	☐
4) Protecting the baby from infection	☐
	☐
5) Not known	☐
6) Others	☐

आर.डी.गार्डी चिकित्सा महाविद्यालय उज्जैन (म.प्र)
स्वास्थ्य कर्मियों के लिए नवजात शिशु की देखभाल हेतु प्रश्नावली

खंड-क: सामान्य जानकारी

**1. आपकी उम्र क्या है?**	______________(साल)
**2. आप कितने वर्षो से प्रैक्टिस कर रहे है?**	______________ (साल)
**3. आप अपनी प्रैक्टिस में एक दिन में कितने मरीज देखते है?**	
1. 5-10	☐
2. 10-20	☐
3. 20-40	☐
4. >50	☐
**4. क्या आप अपने प्रैक्टिस में बच्चो को भी देखते है?**	
हां	☐
नहीं	☐

खंड-ख: नवजात शिशु देखभाल अभ्यास (केवल एक चिन्ह लगा सकते है.)

**5. जन्म के किनते समय बाद स्तनपान शुरू करना चाहिए?**	
1. 1आधे घंटे के अन्दर	☐
2. 2.जन्म के 1-6 घंटे के बाद	☐
3. 6-12 घंटे के बाद	☐
4. जन्म के 12 घंटे के बाद	☐
5. पता नहीं	☐
6. अन्य	______________
**6. एक माँ को कब तक अपने शिशु को केवल अपना दूध पिलाना चाहिए?**	
1. एक माह	☐
2. दो माह	☐
3. 4 माह	☐
4. 6 माह	☐
5. पता नहीं	☐

6. अन्य	______________
**7. आपके अनुसार एक माँ को स्तानपान कब पुर्णतः बंद कर देना चाहिए?**	
1. 6 माह	☐
2. 12 माह	☐
3. 18 माह	☐
4. 24 माह	☐
5. 2 वर्ष बाद	☐
6. पता नहीं	☐
7. अन्य	______________
**8. क्या आप जानते है की राष्ट्रीय गाइड लाइन के अनुसार नवजात शिशु को विटामिन "K" की कितनी खुराक देते है?**	
1. 0.5 मि.ग्रा	☐
2. 1 मि.ग्रा	☐
3. 5 मि.ग्रा	☐
4. 10 मि.ग्रा	☐
5. पता नहीं	☐
6. अन्य	______________
**9. प्रसव के पश्चात नवजात शिशु की नाभि की देखभाल करने का सर्वोत्तम तरीका क्या है?**	
1. सूखने के लिए छोड़ देंगे	☐
2. साबुन और पानी से नियमित साफ़ करेंगे	☐
3. एंटीबायोटिक पाउडर लगायेंगे	☐
4. अस्पताल भेजेंगे	☐
5. पता नहीं	☐
6. अन्य	______________
**10. आपके अनुसार नवजात शिशु में तापमान को नियंत्रित करने का सबसे अच्छा तरीका क्या है?**	
1. शिशु को गुनगुने पानी से नहलाना	☐
2. शिशु को कपडे पहनना और सिर को ढकना	☐
3. शिशु को उसकी माँ की त्वचा से चिपकाकर रखना	☐
4. शिशु को $28\text{-}30^0$ तापमान वाले कमरे में रखना	☐
5. शिशु को ऊष्मा के पास रखना जैसे: हीटर, आग इत्यादि	☐
6. पता नहीं	☐

7. अन्य	____________
**11. जन्म के समय कम वजन वाले शिशु को आप किस तरह परिभाषित करेंगे?**	
1. 3000 ग्राम से कम	☐
2. 2500 ग्राम से कम	☐
3. 1500 ग्राम से कम	☐
4. 1000 ग्राम से कम	☐
5. पता नहीं	☐
6. अन्य	____________
**12. क्या आप बच्चों का वजन लेते है**	
1. हां	☐
2. नहीं	☐
**13. क्या आपको लगता है की बच्चों को उनके वजन के अनुसार दवाइयाँ देनी चाहिए?**	
1. हां	☐
2. नहीं	☐
**14. क्या आप बच्चों के टीकाकरण के बारें में पूछते है?**	
1. हां	☐
2. नहीं	☐

<u>खंड-ग: नवजात शिशु देखभाल अभ्यास (एक से अधिक उत्तरों पर चिन्ह लगा सकते है.)</u>

**15. यदि प्रसव के कुछ दिनों के अन्दर माता अनुभव करती है की उसे पर्याप्त मात्रा में स्तन से दूध नहीं आरहा है, तो आप उसे क्या सलाह देंगे?**	
1. उपरी दूध दे (बोतल द्वारा)	☐
2. बार-बार स्तनपान कराएँ	☐
3. जब तक दूध शुरू न हो तब तक उसे गूढ़ का पानी, प्राकृतिक तरल पदार्थ या शहद का पानी दे	☐
4. माँ को सलाह दे की उसके बच्चे को कोई और माँ स्तनपान कराये उदा: परिवार,पडोसी जिसे पर्याप्त मात्रा में स्तन दूध हो	☐

5. पता नहीं	☐
6. अन्य	______________
**16. ऐसे नवजात शिशु जिसकी स्वशन डर असामान्य हो आप ऐसे शिशु की देखभाल कैसे करेंगे?**	
1. शिशु को कपडे से पोछना	☐
2. बेग और मास्क की सहायता से शिशु को स्वशन में मदद करना	☐
3. आवश्यकता होने पर नाक और मुंह को साफ करना	☐
4. शिशु को उल्टा करके थपथपाना	☐
5. शिशु पर ठंडा पानी डालना	☐
6. पता नहीं	☐
7. अन्य	______________
**17. नवजात शिशु में अत्यधिक रक्त प्रवाह को रोकने के लिए क्या करना चाहिए?**	
1. शिशु को स्तनपान कराना	☐
2. किसी भी दवाई की जरुरत नहीं है	☐
3. विटामिन “K” देना	☐
4. पता नहीं	☐
5. अन्य	______________
**18. कम वजन वालें शिशु की देखभाल में कौन से उपाय महत्वपूर्ण है?**	
1. शिशु को बार–बार नहलाना	☐
2. जल्द से जल्द एवं बार-बार स्तनपान कराना	☐
3. शिशु को गर्म बनाये रखना	☐
4. संक्रमण होने से बचाना	☐
5. पता नहीं	☐
6. अन्य	______________
